# Active Sites of Reduced Epidermal Fluorescence1 (REF1) Isoforms Contain Amino Acid Substitutions That Are Different between Monocots and Dicots

**DOI:** 10.1371/journal.pone.0165867

**Published:** 2016-10-31

**Authors:** Tagnon D. Missihoun, Simeon O. Kotchoni, Dorothea Bartels

**Affiliations:** 1 Department of Biology, Rutgers University, Camden, New Jersey, United States of America; 2 Center for Computational and Integrative Biology, Rutgers University, Camden, New Jersey, United States of America; 3 Institute of Molecular Physiology and Biotechnology of Plants (IMBIO), University of Bonn, Bonn, Germany; Institute for Sustainable Plant Protection, C.N.R., ITALY

## Abstract

Plant aldehyde dehydrogenases (ALDHs) play important roles in cell wall biosynthesis, growth, development, and tolerance to biotic and abiotic stresses. The Reduced Epidermal Fluorescence1 is encoded by the subfamily 2C of ALDHs and was shown to oxidise coniferaldehyde and sinapaldehyde to ferulic acid and sinapic acid in the phenylpropanoid pathway, respectively. This knowledge has been gained from works in the dicotyledon model species *Arabidopsis thaliana* then used to functionally annotate ALDH2C isoforms in other species, based on the orthology principle. However, the extent to which the ALDH isoforms differ between monocotyledons and dicotyledons has rarely been accessed side-by-side. In this study, we used a phylogenetic approach to address this question. We have analysed the *ALDH* genes in *Brachypodium distachyon*, alongside those of other sequenced monocotyledon and dicotyledon species to examine traits supporting either a convergent or divergent evolution of the ALDH2C/REF1-type proteins. We found that *B*. *distachyon*, like other grasses, contains more ALDH2C/REF1 isoforms than *A*. *thaliana* and other dicotyledon species. Some amino acid residues in ALDH2C/REF1 isoforms were found as being conserved in dicotyledons but substituted by non-equivalent residues in monocotyledons. One example of those substitutions concerns a conserved phenylalanine and a conserved tyrosine in monocotyledons and dicotyledons, respectively. Protein structure modelling suggests that the presence of tyrosine would widen the substrate-binding pocket in the dicotyledons, and thereby influence substrate specificity. We discussed the importance of these findings as new hints to investigate why ferulic acid contents and cell wall digestibility differ between the dicotyledon and monocotyledon species.

## Introduction

In plants, the superfamily of the aldehyde dehydrogenases (ALDHs) is generally comprised of several protein families and sub-families, each with differing roles in plant growth and development or responses to biotic and/or abiotic stresses [1,2]. Maize mitochondrial ALDH2B2 is the nuclear restorer of cytoplasmic male sterility [3,4]; rice mitochondrial ALDH family 2 is thought to be essential for the detoxification of acetaldehyde during re-aeration after submergence [5]; whereas OsALDH7B6 from rice is required for seed maturation and maintenance of seed viability through the detoxification of aldehydes generated by lipid peroxidation [6]. Transcripts of several plant *ALDH* genes increase in response to environmental stresses such as dehydration, salinity, excessive light or wounding [7–16]. The aldehyde dehydrogenase ALDH1 isolated from the plant *Artemisia annua* had more than 60% amino acid sequence identity with the subfamily 2C of ALDHs in rice and maize, and catalyzed the oxidation of dihydroartemisinic aldehyde into dihydroartemisinic acid [17]. ALDHs were thus shown to be involved in the biosynthesis of artemisinin in plants [18,19]. Nair et al. [20] have shown that the *reduced epidermal fluorescence1* (*ref1*) phenotype (characterised by a reduced cell wall strength and an accumulation of less than 30% of the sinapate esters found in the wild type) of *Arabidopsis thaliana* is caused by a mutation in *ALDH2C4* (AT3G24503), and that AtALDH2C4/REF1 is important for oxidizing coniferaldehyde and sinapaldehyde into ferulic acid and sinapic acid, respectively, in the phenylpropanoid pathway. Ferulic acid is a hydroxycinnamic acid which, in commelinid monocots, particularly grasses, is ester- and ether-linked to the cell wall polymers of glucuronoarabinoxylan and to lignin, respectively [21,22], whereas in dicots, it is associated with pectic polysaccharides via ester linkages ([23], and references therein). Moreover, ferulic acid can oxidatively cross-link to form covalent ether bonds or C–C bonds between chains of polysaccharides and lignin. Polysaccharides thus become less accessible to degradative enzymes. Consistent with this, several biochemical and genetic studies have established that the ferulic acid content is negatively correlated with the cell wall digestibility in forage grasses and crops [23–30]. All of these studies underline the functional diversity of ALDH proteins, mirrored by the number of *ALDH* genes generally found in plant species, and their implication in the cell wall structure.

Sequenced plant genomes show ALDH families and sub-families of various gene numbers, each protein with enzymatic properties that may be similar, overlapping or different [2]. Based on the orthology principle, ALDH isoforms have often been functionally annotated in plants according to established information for Arabidopsis [2,31]. However, comparative examination of enzymatic properties of ALDH isoforms in monocotyledon plants relative to dicotyledon plants is lacking.

In this study, we have analysed the *B*. *distachyon ALDH* genes, alongside those of other sequenced monocotyledon and dicotyledon species, to investigate traits supporting either a convergent or divergent evolution of plant ALDH functions. To examine how multiplicity and sequence diversity of isoforms between monocotyledon and dicotyledon plants would influence ALDH enzymatic activity we used the *ALDH2C* subfamily of genes, as their greater specificity towards aromatic aldehyde substrates has been well established compared to the other subfamilies in plant [20,32]. Our results indicate that the *B*. *distachyon* genome contains members of the plant-specific ALDH families. Although the ALDH sub-families 2 and 3 are generally represented by more than 3 gene isoforms among plant species, we found a low level of polymorphism between the ALDH2C/REF1-type protein sequences in dicotyledon and monocotyledon plants. One such polymorphism is a conserved phenylalanine residue within the active site of the monocotyledon sequences that, in the conserved dicotyledon sequences, is substituted by a tyrosine residue. Even though this substitution can be viewed as a conserved amino acid substitution, protein structure modelling suggests that the substitution will result in an enlargement of the substrate-binding site, thus altering the substrate specificity of the dicotyledon ALDH2C/REF1 isoforms. Our data, therefore, suggest a difference in substrate specificity of coniferaldehyde/sinapaldehyde dehydrogenases between monocotyledon and dicotyledon plants, which to some extent, may contribute to the different levels of ferulic acid content and cell wall digestibility between dicotyledon and monocotyledon plants.

## Materials and Methods

### Identification and annotation of Brachypodium ALDH proteins

The genome sequence of *Brachypodium distachyon* line Bd21 deposited in the PHYTOZOME v10.2 database (http://www.phytozome.org) was used. ALDH amino acid sequences from rice (*Oryza sativa*), maize (*Zea mays*), and Arabidopsis (*Arabidopsis thaliana*) [2] were retrieved from the database and used to search for Brachypodium *ALDH* sequences by BLASTP with default settings [33] (S2 Table). The presence of characteristic sequence domains within ALDH proteins was verified in the retrieved sequences: PF00171 (ALDH domain), PS00070 (ALDH cysteine active site), PS00687 (ALDH glutamic acid active site), KOG2450 (aldehyde dehydrogenase), KOG2451 (aldehyde dehydrogenase), KOG 2453 (aldehyde dehydrogenase), and KOG2456 (aldehyde dehydrogenase). After removing redundant sequences, the resulting, deduced Brachypodium ALDH protein sequences were annotated using guidelines established by the ALDH Gene Nomenclature Committee (AGNC) [34], wherein, proteins with more than 40% amino acid sequence identity were grouped in a family, and sequences with more than 60% identity composed a subfamily. Amino acid sequences with less than 40% identity would describe a new ALDH protein family.

### Phylogenetic analysis

The protein sequences of ALDH genes in *A*. *thaliana* [31], *Eutrema salsugineum* [35], *Gossypium raimondii* [36], *Glycine max* [37], *Populus trichocarpa* [2], *O*. *sativa* [38], *Sorghum bicolor* [2], *Setaria italica* [39], and *Z*. *mays* [40] were all retrieved from the PHYTOZOME v10.2 database. The maize gene RF2E (Genebank accession: KM225858) was missing in the PHYTOZOME database (V10.2) and was not includedin the analysis because it was truncated and would translate into an incomplete ALDH protein. Multiple alignments of ALDH protein sequences were carried out using MUSCLE in MEGA6 software with default settings [41]. Resulting alignments were later edited using BioEdit V7.2.5 [42] (http://www.mbio.ncsu.edu/BioEdit/bioedit.html). Phylogenetic trees were constructed with MEGA6 software [41,43] using Maximum Likelihood method and General Reverse Transcriptase model (rtREV) with a Gamma distributed with Invariant sites (G+I) model. Gaps were handled with the ‘Partial deletion’ option and 95% site coverage cut-off. The tree topology was validated by 1000 bootstrap replications and the tree was unrooted. Sequence statistics were performed in MEGA6.

### Prediction of protein structures

The effects of amino acid substitutions in the ALDH proteins were predicted from the crystal structure of the maize RF2C (ZmALDH2C1) protein (Protein Data Base number: 4PXL). All mutational analyses were performed using DeepView/Swiss-PDBViewer v4.1 software (http://www.expasy.org/spdbv/) [44,45]. Several possible conformations were examined for each mutant residue, with conformations producing the least number of clashes with adjacent residues being pre-selected. Energy minimization was performed after selection with GROMOS96 [46] implemented in DeepView/Swiss-PDBViewer. The Ramachandran Plot was used to identify the most allowed conformations containing mutant residues.

## Results

### The ALDH protein superfamily in *B*. *distachyon*

*Brachypodium distachyon* is a monocotyledon species that is closely related to crop plants such as, rice (*Oryza sativa*), wheat (*Triticum aestivum*), barley (*Hordeum vulgare*), rye (*Secale cereale*), and oats (*Avena sativa*) [47–52]. We have identified a total of nineteen *ALDH* genes in the *B*. *distachyon* genome (release version v2.1; PHYTOZOME v10.2). The genes (*BdALDHs*) can be grouped into ten families following the ALDH Gene Nomenclature Committee (AGNC) criteria (Table 1). Based on these criteria, the assignment of the individual BdALDH to a family and a subgroup was performed based on the percentage of identity amino acid residues: a minimum of 40% amino acid identity for the proteins of the same family, and a minimum of 60% amino acid identity for the proteins of the subgroups. The percentage of amino acid identity was obtained from a BLASTP search using the ALDH amino acid sequences from rice (*Oryza sativa*), maize (*Zea mays*), and Arabidopsis (*Arabidopsis thaliana*) as queries to search in the genome sequence of *B*. *distachyon*. Each gene was assigned the root symbol ‘ALDH’ followed by the family designation number (1, 2, 3, etc.), the subfamily identifier (A, B, C, D, etc.), and the individual gene number. The largest families are 2 and 3, composed of four and five genes respectively. Families 10 and 18 contain two genes each, with only one gene in each of families 5, 6, 7, 11, 12, and 22.

**Table 1:** Aldehyde dehydrogenase (ALDH) protein families in *Brachypodium distachyon*.

Family name	Gene name	Phytozome ID	Accession number (NCBI)	Number of amino acids
**Family 2**	BdALDH2B1	Bradi1g43770	XP_003563967.1	548
	BdALDH2C1	Bradi2g42360	XP_003569290.1	500
	BdALDH2C2	Bradi2g42380	XP_003569291.1	504
	BdALDH2C5	Bradi1g37090	XP_003563695.1	509
**Family 3**	BdALDH3E1	Bradi3g50180	XP_003569983.1	485
	BdALDH3E2	Bradi5g17110	XP_003580245.1	495
	BdALDH3E3	Bradi3g50200	XP_003569985.1	484
	BdALDH3H1	Bradi4g41190	XP_010238657.1	479
	BdALDH3H2	Bradi4g23620	XP_003577758.1	481
**Family 5**	BdALDH5F1	Bradi3g05490	XP_003570336.1	529
**Family 6**	BdALDH6B1	Bradi1g54940	XP_003557446.1	537
**Family 7**	BdALDH7B6	Bradi4g31310	XP_003578181.1	509
**Family 10**	BdALDH10A5	Bradi5g12617	XP_003579919.1	506
	BdALDH10A9	Bradi3g36150	XP_003574495.1	501
**Family 11**	BdALDH11A3	Bradi3g36930	XP_003574540.1	502
**Family 12**	BdALDH12A1	Bradi2g18550	XP_010231104.1	551
**Family 18**	BdALDH18B1	Bradi2g23507	XP_003568327.1	793
	BdALDH18B2	Bradi2g54920	XP_003564608.1	732
**Family 22**	BdALDH22A1	Bradi1g17080	XP_003562417.1	594

**Fig 1:**
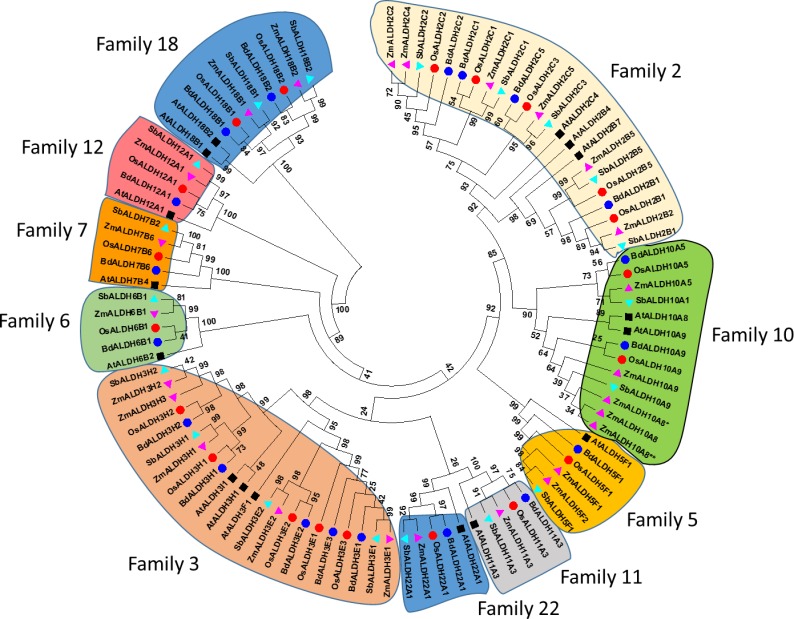
Phylogenetic analysis of the ALDH superfamily in selected monocotyledonous species. Sequences of the dicotyledon *A*. *thaliana* were used as outgroup. Sequences were aligned by using MUSCLE, and the unrooted phylogram was generated by using Maximum Likelihood statistical method (MEGA6 software). Bootstrap values from 1000 replicates are indicated at each branch. Prefixes and symbols were used to indicate the origin of the sequences: At and black square, *Arabidopsis thaliana*; Bd and blue circle, *Brachypodium distachyon*; Os and red circle, *Oryza sativa*; Sb and cyan upward triangle, *Sorghum bicolor*; Zm and magenta downward triangle, *Zea mays*. Asterisks (* and **) added to ZmALDH10A8 within the box of family 10 refer to the loci GRMZM2G146754 (Phytozome v10.2) and AC74867.1 (NCBI), respectively.

Using Arabidopsis ALDH sequences as out-group sequences the relationship of the *Bd*ALDHs to orthologs in other grass species was examined (Fig 1). BdALDHs group together with those of rice, sorghum, and maize according to their assigned families and sub-families. In most cases, the BdALDH sequences aligned very closely with rice sequences more than to the other grasses. In each family, the Arabidopsis ALDHs formed a separate group outside of the cluster of monocotyledon sequences. All ALDH families commonly found in plants, including those specific to plants, were identified in *B*. *distachyon*.

### ALDH sequence polymorphisms and functional diversity

**Fig 2:**
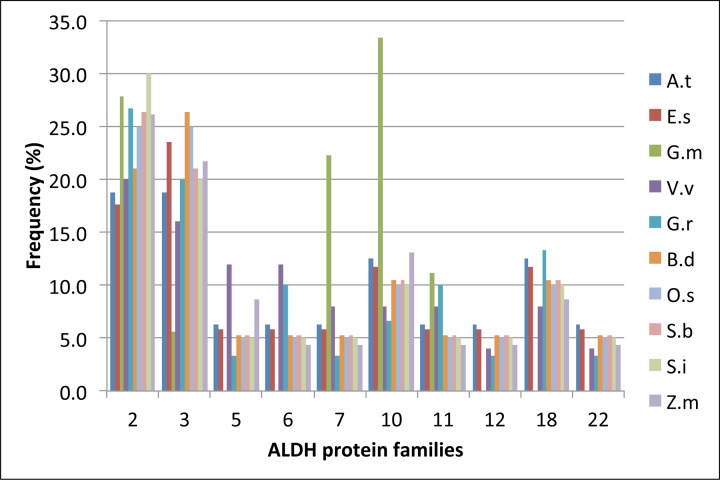
Frequencies of plant ALDH families within selected dicotyledonous and monocotyledonous species. Abbreviations and their corresponding species names: A.t: *Arabidopsis thaliana*; E.s: *Eutrema salsugineum*; G.m: *Glycine max*; V.v: *Vitis vinifera*; G.r: *Gossypium raimondii*; B.d: *Brachypodium distachyon*; O.s: *Oryza sativa*; S.b: *Sorghum bicolor*; S.i: *Setaria italica*; Z.m: *Zea mays*. Note that the ALDH families 5, 6, 12, 18, 22 were not found in *Glycine max* (G.m) [37]. The bar representing *G*. *max* is therefore not displayed for those missing families in the plot.

Our analysis of *BdALDHs* indicates that families 2 and 3 have four and five members respectively, and they are more polymorphic than the other *BdALDH* families with only one or two genes in each family (Fig 1). Furthermore, if the number of members in each *ALDH* family in different monocotyledon and dicotyledon plants is expressed as the ratio of number of genes in the family to total number of *ALDH* genes in the species, the ratios show that families 2 and 3 are consistently the largest polymorphic families with the exception of soybean (*Glycine max*) (S1 Table; Fig 2).

**Fig 3:**
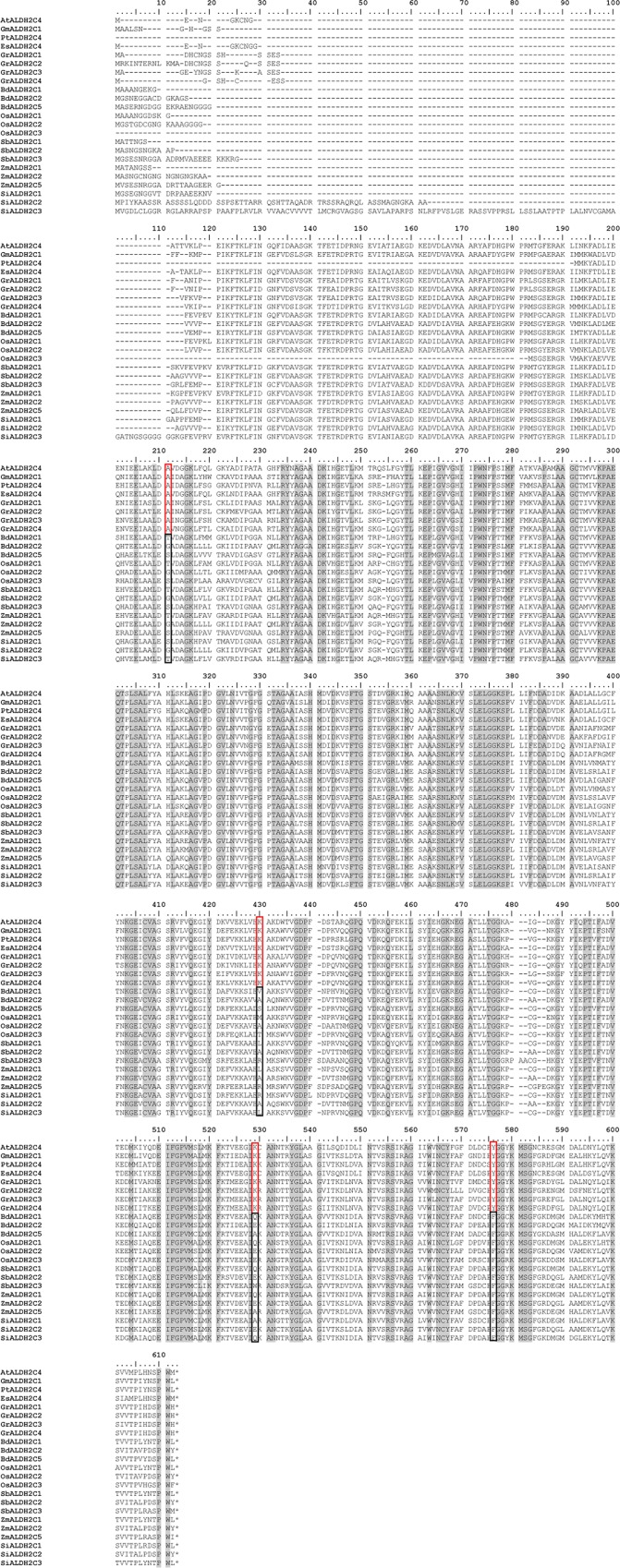
Alignment of the amino acid sequences of ALDH2C proteins. Sequences were retrieved from PHYTOZOME v10.2 and aligned by MUSCLE implemented in MEGA6. Identical residues are shown in a grey-shaded background. Sites of lineage-specific polymorphism (see text for detail) are outlined: red and black for the dicotyledonous and the monocotyledonous sequences, respectively. Prefixes were used to differentiate between genes from species: At, *Arabidopsis thaliana*; Gm, *Glycine max*; Pt, *Populus tricocarpa*; Es, *Eutrema salsugineum*; Gr, *Gossypium raimondii*; Bd, *Brachypodium distachyon*; Os, *Oryza sativa*; Sb, *Sorghum bicolor*; Zm, *Zea mays; Si*, *Setaria italica*. Asterisks (*) indicate the stop codon.

In plants, the protein family 2 is composed of the subfamilies 2B and 2C. Based on analyses of Arabidopsis loss-of-function mutants, the proteins of the subfamily 2C (ALDH2C/REF1-type proteins) catalyse the oxidation of coniferaldehyde and sinapaldehyde to ferulic acid and sinapic acid, respectively [20]. Arabidopsis, like most dicotyledons with published *ALDH* gene families (*Eutrema salsugineum*, *Glycine max*, *Vitis vinifera*, *Gossypium raimondii*), contains only one or two *ALDH2C/REF1*-type genes compared to four *ALDH2C/REF1*-type genes in *B*. *distachyon* and other monocotyledons (Table 1; Fig 1). Based on the orthology principle, ALDH isoforms have often been functionally annotated in plants according to established information in Arabidopsis but the degree of *ALDH* sequence diversity and of its effect on the specificity of function has rarely been assessed among species. To determine the degree of divergence between *ALDH2C* genes in dicotyledons and monocotyledons the protein amino acid sequences were aligned (Fig 3; S3 Table) then nucleotide divergence per pair of *ALDH2C* sequences was determined within and between selected species by using MEGA6. The nucleotide divergence between isoforms of the same lineage (within-group divergence) was similar within dicotyledons (0.33 ± 0.01) and monocotyledons (0.28 ± 0.01). In contrast, the nucleotide divergence between the two lineages was significantly higher (0.52 ± 0.02) than within the groups. To verify whether this difference has arisen either by chance or selection, we ran the codon-based Z-test of purifying selection implemented in MEGA6 with a null hypothesis of strict neutrality [41]. The null hypothesis was rejected in favour of the alternative hypothesis of purifying selection within the ALDH2Cs in both lineages (*P* < 0.0001). Though, the null hypothesis could not be rejected in favor of the alternative hypothesis of positive selective. These data indicated that the number of synonymous substitutions per synonymous site (dS) was significantly superior to the number of non-synonymous substitutions per non-synonymous site (dN) within ALDH2Cs in both lineages.

We examined the selection of each amino acid from the alignment shown in Fig 3. As noted above with the codon-based Z-test, nearly all sites were found to be under a purifying selection. Only 14 (3%) out of the 423 codons showed a ratio of dN/dS > 1 compared to 395 (93%) out of 423 codons that had a dN/dS < 1.

Given the fact that the previous two approaches tend to detect hotspots of positive selection more efficiently than they did for the sites of mid-level positive or negative selection, we used the Tajima’s *D* statistics to gain better insight into the pattern of nucleotide variations. We found *D* = 2.71 (θ = 0.17, π = 0.29, *P* < 0.001), which suggests that nucleotide variation within ALDH2Cs might overall be less frequent than expected but a few alleles of nucleotide polymorphisms would be present at high frequency among the species.

### Nucleotide composition, amino acid content, and codon usage are biased among ALDH2C isoforms

**Fig 4:**
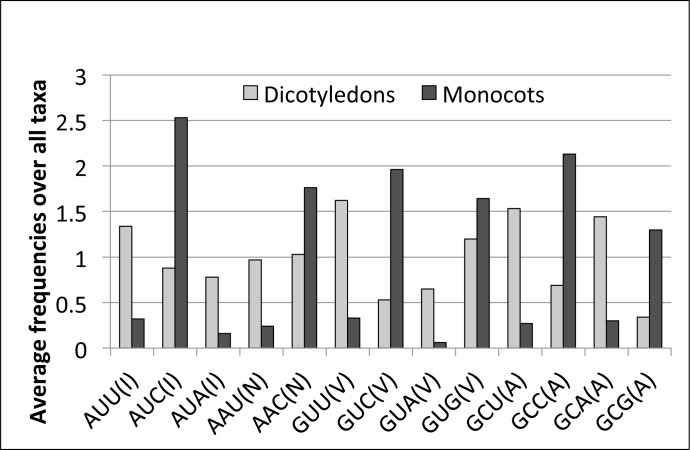
Relative synonymous codon usage. Estimates were based on the protein coding sequences of 8 and 15 dicotyledonous and monocotyledonous ALDH2C/REF1 isoforms, respectively. Letters in brackets represent amino acids. I: isoleucine; N: asparagine; V: valine; A: alanine.

Characterisation of the nucleotide and amino acid residue composition between *ALDH2C* isoforms showed that dicotyledon *ALDH2C/REF1* coding sequences are enriched in A and T, specifically, at the first and third positions of codons (Table 2). In contrast, monocotyledon sequences were significantly enriched in C and G at these positions in the codon, especially G in position 3. The dicotyledon sequences have a significantly high proportion of the two amino acid residues Ile and Asn, whereas the monocotyledon sequences have a significantly high proportion of Ala and Val (Table 3). In reviewing the nucleotide composition of the codons for these amino acids, we found that codons with A or T in the third base position were more frequently used for Ile and Asn in dicotyledon ALDH2C sequences than in monocotyledon sequences, whereas C or G in the third base position was the preferred nucleotide for Ala and Val in monocotyledon ALDH2C sequences (Fig 4). These data suggested the presence of some lineage-specific amino acid polymorphisms within ALDH2C/REF1 sequences in the dicotyledon and monocotyledon species.

**Table 2:** Frequency of nucleotides in codons of ALDH2C proteins.

	Position 1				Position 2				Position 3			
	T-1	C-1	A-1	G-1	T-2	C-2	A-2	G-2	T-3	C-3	A-3	G-3
**Dicots**	18.1 ± 1.1	15.7 ± 1.5	36.9 ± 2.0	29.3 ± 1.1	23.9 ± 1.3	26.9 ± 1.3	35.4 ± 2.2	13.8 ± 0.5	32.6 ± 2.7	21.4 ± 3.3	26.5 ± 1.3	19.5 ± 1.1
**Monocots**	10.5 ± 1.0	21.4 ± 1.6	29.8 ± 2.4	38.2 ± 2.3	23.0 ± 1.2	27.9 ± 1.5	30.7 ± 3.4	18.4 ± 4.4	6.5 ± 2.3	50.9 ± 4.0	6.3 ± 2.0	36.3 ± 2.2
*P*-value	**1.9E-13**	**3.0E-08**	**4.9E-07**	**8.5E-10**	0.11	0.107	0.002	0.003	**5.9E-17**	**4.5E-14**	**1.3E-17**	**3.0E-15**

Parsimony informative sites based on the alignment of nucleotides within the protein coding regions of both dicotyledonous and monocotyledonous *ALDH2C* genes were used in the calculation. Values in the table indicate frequency average ± standard errors. *P*-values were obtained after the Student’s *t* test. Only *P*-values < 0.001 (showed in bold characters) were considered significant.

**Table 3:** Frequency of amino acids within the ALDH2C/REF1 isoforms among dicotyledons and monocotyledons.

	Dicotyledonous (Average ± SE)	Monocotyledonous (Average ± SE)	*P*-value (Student’s *t* test)
**Alanine**	9.74 ± 0.75	**11.81 ± 1.01**	6.21E-05*
Cysteine	1.17 ± 0.34	1.03 ± 0.39	0.428
Aspartic acid	6.02 ± 0.92	6.74 ± 0.60	0.038
Glutamic acid	6.45 ± 0.63	6.66 ± 0.86	0.545
Phenylanine	4.97 ± 0.49	4.55 ± 0.57	0.097
Glycine	4.09 ± 0.34	5.00 ± 0.73	0.003
Histidine	1.86 ± 0.33	1.62 ± 0.36	0.142
**Isoleucine**	**9.48 ± 0.32**	5.53 ± 1.05	1.346E-09*
Lysine	8.52 ± 0.55	6.86 ± 1.43	0.005
Leucine	8.00 ± 0.61	7.21 ± 0.61	0.008
Methionine	3.67 ± 0.38	3.99 ± 0.57	0.167
**Asparagine**	**3.71 ± 0.98**	2.12 ± 0.78	4.64E-04*
Proline	2.19 ± 0.47	2.79 ± 0.55	0.019
Glutamine	2.48 ± 0.32	2.12 ± 0.46	0.069
Arginine	2.90 ± 0.60	4.64 ± 1.70	0.012
Serine	6.70 ± 0.67	5.50 ± 1.40	0.035
Threonine	5.78 ± 0.87	5.82 ± 1.13	0.937
**Valine**	8.39 ± 0.94	**12.40 ± 1.04**	1.49E-08*
Tryptophane	0.30 ± 0.26	0.21 ± 0.17	0.352
Tyrosine	3.59 ± 0.28	3.39 ± 0.83	0.527

Only parsimony informative sites based on the alignment of both dicotyledonous and monocotyledonous ALDH2C amino acid sequences were used in the calculation. Asterisk (*) denotes significance at *P < 0*.*001*. Amino acids that were significantly enriched in dicotyledons or in monocotyledons are shown in bold characters.

### Amino acid substitution analysis

We then compared the amino acids between dicotyledon and monocotyledon ALDH2C sequences and identified 32 sites where residues either strictly differ or overlap between the two plant lineages (Table 4). Overlapping sites were often composed of one amino acid residue common to the two lineages, and between one to five additional variants in one of the lineages, more often in the monocotyledon isoform sequences. Four of the 32 sites (residue number 211, 430, 529 and 576 in Fig 3) were occupied by residues that are consistent within the dicotyledon sequences but different between the monocotyledon isoforms. Amino acid residues at positions 211 and 529 were conserved between *B*. *distachyon* and *Zea mays* isoforms but not at site 430 where there is a mixture of conserved and non-conserved amino acid residues. One of the four sites (residue number 576 in Fig 3) was strictly dimorphic with only two amino acid variants, each conserved in one lineage (Table 5).

**Table 4:** Polymorphic amino acid residues within and between dicotyledonous and monocotyledonous ALDH2C sequences.

Position within the alignment^1^	Dicotyledons	Monocotyledons
**211**	**A**	**T, G, S**
214	A, G	A
264	I	L, I, M, V
275	F	F, Y
309	Y	L, F, V, Y
339	F, Y	F
343	I, V	V
345	K	K, S, M, V, I
362	A	A, S
391	A, V	V
395	A	A, V, S
398	G, A	A, G^2^
406	I	I, V, A
427	L	S, A, L
**430**	**K**	**V, A, S, M, T, L, R**
431	A	A, L, M
442	D	N, D
458	K	K, R
459	I	V, I
461	S	K, R, S, G
464	E	E, D
**529**	**K**	**Q, E, A, W**
533	N, D	N, C, D, S, G
552	T	T, R, M
554	S	T, S, A, V
**576**	**Y**	**F**
579	Y	C, Y, R
586	R	K, R
598	Q	H, Q, A
601	S	S, A, T
605	P	P, A
609	S	T, S

^1^ The positions were identified from the parsimony-informative sites in the alignment showed in Fig 3.

^2^ The amino acid G dominated in the dicotyledon sequences whereas the amino acid A dominated in the monocotyledon sequences at that position. Lines shown in bold contains lineage-specific amino acid variations.

**Table 5:** Comparison of lineage-specific amino acid polymorphisms within the ALDH2C/REF1 isoforms in three selected species.

Position in the alignment^1^	*A thaliana*^2^	*B*. *distachyon*^2^			*Z*. *mays*^2^		
	AtALDH2C4	BdALDH2C1	BdALDH2C2	BdALDH2C5	ZmALDH2C1	ZmALDH2C2	ZmALDH2C5
211	A106	T106	G110	S115	T108	G117	S119
430	K325	V324	A328	S333	L326	A335	R337
529	K418	Q417	E421	A426	Q419	E428	A433
**576**	**Y465**	**F464**	**F468**	**F473**	**F466**	**F475**	**F480**

^1^ The positions were identified from the parsimony-informative sites in the alignment showed in Fig 3.

^2^ Positions refers to the numbering of amino acid residue within each isoform of species. The strictly dimorphic case of amino acid polymorphisms is shown in bold characters.

While many of the 32 polymorphic sites involved amino acid variants with similar physicochemical properties (Table 4), sites with dissimilar amino acid residues were also identified between the two lineages. For example, L326 and Q419 in ZmALDH2C1 (positions 430 and 529, respectively in Fig 3) are conserved as V324 and Q417 in BdALDH2C1 but in AtALDH2C4 these residues are K325 and K418, respectively; showing a non-polar to polar basic amino acid variation at position 430 and a non-polar basic to a polar basic variation at position 529. Furthermore, the active site residue F466 (position 576 in Fig 3), implicated in substrate binding in maize ALDH2C1 (RF2C: [32]) and conserved in all monocotyledon isoforms examined in this study is a tyrosine, a polar amino acid, in all of the dicotyledon sequences (Fig 3). Overall, about 10% of the amino acid residues were found to be different between monocotyledon and dicotyledon ALDH2C/REF1 isoforms. The physicochemical properties of the concerned amino acids were similar or conserved between the two lineages. We examined whether those substitutions would influence the tridimensional structure of the ALDH2C isoforms.

### Possible effects of amino acid variations on the catalytic properties of maize RF2C (ZmALDH2C1) protein

**Fig 5:**
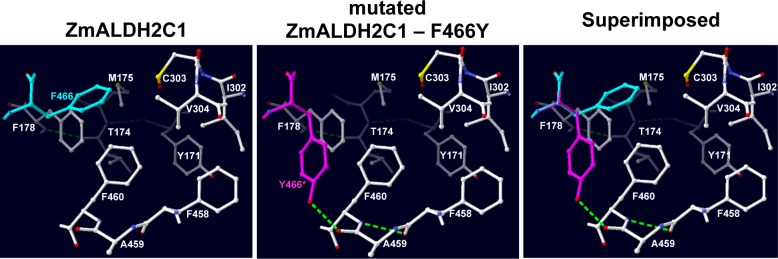
A slab view of the substrate channel of ZmALDH2C1 from the bottom (left panel). A similar view of the channel after the mutation F466Y to Y466 is shown in the middle panel. A superimposed picture of the two views is shown in the right panel. The crystal structure of ZmALDH2C1 (PDB file: 4PXL) was retrieved from the Protein Data Bank and used to analyse the effects of the mutation on the protein conformation. Residues F466 and Y466 are shown in cyan and magenta colors, respectively. Oxygen, nitrogen, and sulfur atoms are shown in red, blue, and yellow, respectively. Green discontinued lines represent H-bonds. The most plausible arrangement of the Y466 is shown (see text for details). Based on this arrangement, the mutation F466Y appears to widen the substrate channel and to create new H-bonds in its vicinity. Model manipulation and mutation analysis were performed in the Swiss-Pdb Viewer software V4.1.

Using the published crystal structure of the maize ZmALDH2C1 (RF2C) protein [32] we predicted the effect on enzymatic activity of exchanging the conserved monocotyledon amino acid with its alternative dicotyledon (Arabidopsis) amino acid. The residue Q419 (position 529 in Fig 3) in ZmALDH2C1 is located on the external surface of the protein and therefore the exchange Q419K was predicted to have no or minor impact on enzyme topology. However, residues T108 (position 211 in Fig 3) and L326 (position 430 in Fig 3) are both located inside of the protein and consequently the exchanges T108A and L326K are predicted to disrupt some existing H-bonds as well as participate in the formation of new H-bonds between nearby or distant residues. Similar predictions were made for the F466Y exchange within the substrate-binding site (Fig 5). The substrate-binding pocket is composed primarily of aromatic and non-polar residues [32] and during modelling several possible configurations with the exchange residue Y466 were analysed. Most of the predicted configurations induce clashes with adjacent residues and were therefore not retained. One of the most likely permitted conformations with Y466 implies the displacement of the polar side-chain toward the surface of the protein, and away from the hydrophobic microenvironment of the substrate-binding site, resulting in a widening of the substrate-binding channel (Fig 5). We suggest that this monocotyledon to dicotyledon exchange of amino acid residues could explain some of the substrate specificity observed between the ALDH2C family isoforms.

## Discussion

ALDH enzymes are found in almost all organisms and they are expressed in diverse organs and tissues where they play diverse roles [1,2]. Besides the effect of speciation, they appear to evolve through gene duplication as shown in diverse species [31,36,37,39]. Although the driving forces of the duplication events are unclear, it is remarkable that the *ALDH* genes often do not have the same expression patterns and a number of isoforms per family. We found that *B*. *distachyon*, like other species, contains more isoforms within the ALDH2 and ALDH3 families than in the other families. This suggests that *ALDH* genes might have evolved to fulfill different functions. In this scenario, distinctive features within the sequences of evolved *ALDH* genes would support their functional specialisation. Alternatively, the duplicated genes might have randomly evolved and therefore would not impact on the primary function or biochemical properties of the enzymes. To understand the evolutionary pattern of the duplicated *ALDH* genes within a subfamily, we used the ALDH2C amino acid sequences to test whether independent ALDH isoforms of genetically distant species, i.e. monocotyledons and dicotyledons, contain similar structural changes, and if so, whether those structural changes are likely to alter the enzyme properties such as substrate specificity. Because proteins are grouped in a subfamily based on at least 60% sequence conservation (AGNC recommendations), such a comparison might be biased. We were looking for sequence features within the remaining 40% of sequences, which might differ between genetically distant isoforms and perhaps between isoforms of a given single species. Our focus on the ALDH2C subfamily was guided by their high specificity toward coniferaldehyde and sinapaldehyde, as shown by Nair et al [20], and by their involvement in the biosynthesis of ferulic acid. Those features are unique to this subfamily, and they will be valuable for the future physiological and biochemical studies. We found that the analysed ALDH2C sequences were most likely suggested to a negative selection. This observation is significant when considering that monocotyledons and dicotyledons might have diverged 340 million years ago [53]; it suggests that recently evolved ALDHs have retained the ancestral enzymatic property, which is to oxidise aldehyde molecules to their corresponding carboxylic acids. In agreement with this, previous studies showed that the ALDH2C/REF1 proteins oxidise coniferaldehyde and sinapaldehyde to ferulic acid and sinapic acid, respectively, whereas ALDH2B proteins preferably oxidise acetaldehyde to acetic acid and would be involved in pollen fertility and aerobic fermentation [3,4,5,20,32,54]. However, besides the preferred substrates, *in vitro* enzymatic tests showed that ALDH2C and ALDH2B, as well as other ALDH proteins, also oxidise a range of aldehydes with comparable efficiency [4,6,12,32,55]. This leads to the question how ALDH functional specificity is achieved *in planta*.

Our calculation of the Tajima *D* statistics indicated that nucleotide variations within ALDH2Cs were overall low but a few alleles of nucleotide substitutions would be present among the species. This led us to examine whether the substitutions are likely to alter the enzyme substrate specificity. Notably, we found one site within the amino acid sequence alignment that indicates an exchange of a phenylalanine residue in the monocotyledon ALDH2C sequences with a tyrosine in the dicotyledon’s ones. We do not know why the two alleles of that substitution were separately maintained in each lineage (represented by the species analysed in this study), and the biological significance. Our predictions, based on the crystal structure of the maize ZmALDH2C1 protein (RF2C, a homologous protein of ALDH2C4/REF1 in Arabidopsis) [32], suggest that the substitution would widen the substrate-binding pocket of the ALDH2C isoforms in dicotyledons. A similar observation was reported on the comparison of the maize RF2C and RF2F (ZmALDH2C5, homologous to ALDH2C4/REF1 in Arabidopsis) proteins [32]. The authors found that the substrate-binding site of RF2F is much wider because of the presence of V192 and M477 instead of the two aromatic residues F178 and F460 (positions 282 and 570 in the alignment, respectively; Fig 3) in RF2C. They further examined the impacts of these substitutions on the enzymatic activity and found that the cavity width of RF2F correlates with high *K*m values for various substrates most probably due to weaker nonpolar interactions. In contrast, two other isoforms RF2D and RF2E, which do not differ in active site residues, were found to have similar kinetic properties. These findings combined with our current results support the idea that substrate preference and hence specificity among highly conserved ALDH isoforms is defined by a few substitutions within the substrate-binding site of the enzyme. Consistently, examination of duplicated ALDH2 genes in *Drosophila melanogaster* showed that the diameter of the substrate entry channel is restricted by naturally occurring substitutions, which shift substrate specificity among duplicated genes [56,57]. It was demonstrated that eukaryote ALDH1/2s often switched between large and small substrate entry channels after gene duplication, transforming restricted channels into wide opened ones and *vice versa* [58]. We are not aware of any report on a side-by-side comparison of the affinity and catalytic activity of the monocotyledon and dicotyledon ALDH2C-type enzymes toward their preferred substrates coniferaldehyde and sinapaldehyde. But based on those experimental evidences, one may speculate that the exchange of F466Y can potentially alter the specificity of the ALDH2C-type enzymes toward these two substrates because a widened substrate channel is likely to alter the substrate specificity and the activity of the enzyme. Whether that substitution alone can explain why cell walls of monocotyledon species often contain more ferulic acid than the wall of dicotyledon species, however, remains to be examined [21,22]. Indeed, more than one metabolic routes were found to contribute to the ferulic acid content in the plant cell wall. According to de Oliveira et al. [30], the current knowledge suggests that ferulic acid is synthesized from the mainstream phenylpropanoid pathway. In this pathway, L-Phenylalanine is deaminated by phenylalanine ammonia-lyase to produce *t*-cinnamic acid. This step is followed by hydroxylation of the aromatic ring, catalysed by cinnamate 4-hydroxylase, to give *p*-coumaric acid. In the next step, the carboxylic group of *p*-coumaric acid is activated to a thioester via 4-coumarate:CoA ligase to yield *p*-coumaroyl-CoA. This compound is transesterified to shikimate or quinate by the action of *p*-hydroxycinnamoyl CoA:quinate/shikimate *p*-hydroxycinnamoyl-transferase (HCT). The ester is further hydroxylated in the C3 to produce caffeoyl-shikimate/quinate ester by *p*-coumaroyl shikimate/quinate 3-hydroxylase. Caffeoyl-shikimate/ quinate is transesterified back with CoA by HCT and O-methylated in the hydroxyl group in C3 by caffeoyl-CoA O-methyl- transferase (CCoAOMT) to produce feruloyl-CoA, the activated form of ferulic acid [59–61]. Feruloyl-CoA is considered as the major substrate of the enzymes that transfer the ferulic acid moiety into the cell wall by esterification to the cell wall polysaccharides. In a second pathway, feruloyl-CoA is reduced to coniferaldehyde in a reaction catalysed by cinnamoyl-CoA reductase (CCR). Nair et al. [20] showed that coniferaldehyde is oxidised to ferulic acid by ALDH2C4/REF1) in Arabidopsis. In order to be esterified to the cell wall polysaccharides, the free form of ferulic acid must be first activated to its active form feruloyl-CoA [62,63]. The enzyme 4-coumarate:CoA ligase has been demonstrated to be responsible for catalysing the esterification of exogenous-free ferulic acid to feruloyl-CoA *in vivo* [22,64]. In a third possible biosynthetic route, a caffeoyl shikimate esterase [65,66], upstream of feruloyl–CoA, catalyses the conversion of caffeoyl shikimic/quinic acid into caffeic acid that is then O-methylated in the hydroxyl group in C3 by caffeic acid O-methyl- transferase (COMT) to produce ferulic acid. The free ferulic acid may serve as a precursor for the biosynthesis of feruloyl hexose and feruloyl sinapate [67]. Of the three routes, only the second route described above involve the ALDH2C dehydrogenase activity. Recently, an Arabidopsis mutant defective in the *ccr1* gene coding for cinnamoyl-CoA reductase was shown to accumulate significantly higher amounts of ferulic acid compared to the wild type. In contrast, the ferulic acid level was dramatically reduced in a double mutant defective in caffeic acid O-methyltransferase and caffeoyl-CoA 3-O-methyltransferase (*comt ccoaomt*) compared to the wild type [68]. These observations suggest that studying the contribution of each of the three routes to the total ferulic acid pool together with the implications of the active site amino acid substitution described in this study may greatly help develop crops with reduced ferulic acid contents. A way may be found to engineer cell walls with high digestibility based on low ferulic acid content [30]. For now, our current data support the idea that *ALDH* gene duplications did not evolve by pure chance. Their amino acid sequences, albeit showing more than 60% conservation within subfamilies, would include key substitutions that likely confer functional specificity. Biologists often rely on the principle of gene orthology for the transfer of functional information from experimentally characterized genes in model organisms to uncharacterized genes in newly sequenced genomes [69,70]. Our data now calls for caution in this approach.

## Supporting Information

S1 Table Frequencies of plant ALDH families within selected dicotyledonous and monocotyledonous species. (DOC)

S2 Table Aldehyde dehydrogenase proteins used as queries to search for *Brachypodium distachyon* ALDH-coding genes and to build the phylogenetic tree. (XLSX)

S3 Table List of the *ALDH2C* genes used in the analyses. (XLSX)
