# Correction: Active Sites of Reduced Epidermal Fluorescence1 (REF1) Isoforms Contain Amino Acid Substitutions That Are Different between Monocots and Dicots

**DOI:** 10.1371/journal.pone.0200034

**Published:** 2018-06-26

**Authors:** The PLOS ONE Editors

Following publication, concerns were raised regarding the author list and funding disclosures on this article. Officials at Rutgers University-Camden and University of Saskatchewan investigated this matter and agreed that these issues should be corrected as follows:

Peta Bonham-Smith is not included in the author byline and should be listed as the last author. Dr. Bonham-Smith is affiliated with Department of Biology, University of Saskatchewan, Saskatoon, Saskatchewan, Canada. The contributions of this author are as follows: Conceptualization, Formal Analysis, Methodology, Resources, Validation, Writing–review & editing.

The correct citation for this article is: Missihoun TD, Kotchoni SO, Bartels D, Bonham-Smith PC (2016) Active Sites of Reduced Epidermal Fluorescence1 (REF1) Isoforms Contain Amino Acid Substitutions That Are Different between Monocots and Dicots. PLoS ONE 11(10): e0165867. https://doi.org/10.1371/journal.pone.0165867

The Funding Disclosure statement on the published article is incorrect and should declare that this research was supported by Saskatchewan Agriculture Development Fund grant# 20130096.

The affiliation for Tagnon D. Missihoun is incorrectly listed on the published article. While Dr. Missihoun was affiliated with Rutgers University-Camden at the time the manuscript was submitted to *PLOS ONE*, a substantial portion of this research was completed during his tenure at the University of Saskatchewan. Therefore, his affiliation on this published article should be listed as Department of Biology, University of Saskatchewan, Saskatoon, Saskatchewan, Canada. The email addresses listed for Dr. Missihoun on the article are correct.
