# Supplementary material for: Active Sites of Reduced Epidermal Fluorescence1 (REF1) Isoforms Contain Amino Acid Substitutions That Are Different between Monocots and Dicots
Source: PLoS One. 2016 Oct 31;11(10):e0165867. doi: 10.1371/journal.pone.0165867 (PMC5087895; doi:10.1371/journal.pone.0165867)
Supplement: S1 Table — (DOC) [file pone.0165867.s001.doc]

**Supplementary Table 1:** Frequencies of plant ALDH families within selected dicotyledonous and monocotyledonous species

| **Species** | **Families** | **2** | **3** | **5** | **6** | **7** | **10** | **11** | **12** | **18** | **22** |
| --- | --- | --- | --- | --- | --- | --- | --- | --- | --- | --- | --- |
| **Dicotyledons** |  |  |  |  |  |  |  |  |  |  |  |
| *Arabidopsis thaliana* |  | 18.8 | 18.8 | 6.3 | 6.3 | 6.3 | 12.5 | 6.3 | 6.3 | 12.5 | 6.3 |
| *Eutrema salsugineum* |  | 17.6 | 23.5 | 5.9 | 5.9 | 5.9 | 11.8 | 5.9 | 5.9 | 11.8 | 5.9 |
| *Glycine max* |  | 27.8 | 5.6 | 0.0 | 0.0 | 22.2 | 33.3 | 11.1 | 0.0 | 0.0 | 0.0 |
| *Vitis vinifera* |  | 20.0 | 16.0 | 12.0 | 12.0 | 8.0 | 8.0 | 8.0 | 4.0 | 8.0 | 4.0 |
| *Gossypium raimondii* |  | 26.7 | 20.0 | 3.3 | 10.0 | 3.3 | 6.7 | 10.0 | 3.3 | 13.3 | 3.3 |
| **Monocotyledons** |  |  |  |  |  |  |  |  |  |  |  |
| *Brachypodium distachyon* |  | 21.1 | 26.3 | 5.3 | 5.3 | 5.3 | 10.5 | 5.3 | 5.3 | 10.5 | 5.3 |
| *Oryza sativa* |  | 25.0 | 25.0 | 5.0 | 5.0 | 5.0 | 10.0 | 5.0 | 5.0 | 10.0 | 5.0 |
| *Sorghum bicolor* |  | 26.3 | 21.1 | 5.3 | 5.3 | 5.3 | 10.5 | 5.3 | 5.3 | 10.5 | 5.3 |
| *Setaria italica L.* |  | 30.0 | 20.0 | 5.0 | 5.0 | 5.0 | 10.0 | 5.0 | 5.0 | 10.0 | 5.0 |
| *Zea mays* |  | 26.1 | 21.7 | 8.7 | 4.3 | 4.3 | 13.0 | 4.3 | 4.3 | 8.7 | 4.3 |

The frequencies were calculated for each family as the ratio of genes representing the family over the total number of *ALDH* genes found in a given species.
